# Testing the Possible Protective Effect of Ivermectin on Bleomycin-Induced Pulmonary Fibrosis in Albino Rats: Histological and Immunohistochemical Study

**DOI:** 10.3390/medicina62030560

**Published:** 2026-03-18

**Authors:** Eman A. Zaher, Ayman A. Refai, Soha S. Zakaria, Mohammad I. Jumaa, Ala M. Aljehani, Enas Elhosary, Reham A. Al-Dhelaan, Mostafa A. Arafa, Rania H. Elsyade

**Affiliations:** 1 Histology and Cell Biology Department, Faculty of Medicine, Capital University (Formerly Helwan University), Cairo 11795, Egypt; eman.zaher10@med.helwan.edu.eg; 2 Department of Anatomy and Physiology, College of Medicine, Imam Mohammad Ibn Saud Islamic University (IMSIU), Riyadh 13317, Saudi Arabia; 3 Department of Biochemistry, College of Medicine, Imam Mohammad Ibn Saud Islamic University (IMSIU), Riyadh 13317, Saudi Arabia; sshehata@imamu.edu.sa (S.S.Z.); raldhelaan@imamu.edu.sa (R.A.A.-D.); 4 Department of Pathology, College of Medicine, Imam Mohammad Ibn Saud Islamic University (IMSIU), Riyadh 13317, Saudi Arabia; amaljehani@imamu.edu.sa; 5 Pathology Department, Faculty of Medicine, Capital University (Formerly Helwan University), Cairo 11795, Egypt; enas.megahed@med.helwan.edu.eg; 6 The Cancer Research Chair, Department of Surgery, College of Medicine, King Saud University, Riyadh 11461, Saudi Arabia; mostafaarafa@hotmail.com; 7 Department of Epidemiology, High Institute of Public Health, Alexandria University, Alexandria 21526, Egypt; 8 Anatomy and Embryology Department, Faculty of Medicine, Capital University (Formerly Helwan University), Cairo 11795, Egypt; rania.elsyade@med.helwan.edu.eg; 9 Anatomy and Embryology Department, Faculty of Medicine, Galala University, Suez 43511, Egypt

**Keywords:** ivermectin, bleomycin, pulmonary fibrosis, α-SMA, Ki-67, GSH

## Abstract

*Background and Objectives*: Pulmonary fibrosis (PF) is an interstitial lung disease that leads to death and is characterized by excessive collagen deposition and tissue scarring. Bleomycin (BLM) is widely used to induce PF in rodent models, closely resembling human idiopathic pulmonary fibrosis. Ivermectin, a broad-spectrum antiparasitic agent, has recently attracted interest due to its reported anti-inflammatory and antifibrotic effects. The beneficial effects of ivermectin to treat PF may be attributed to suppressing the NLRP3 inflammasome. Ivermectin can cause acute toxicity, including convulsions, when overdosed in animals. In humans, it may induce neurological disorders, particularly in individuals with mutations in the ABCB1 gene. This study aimed to investigate the potential protective role of ivermectin against BLM-induced PF in rats. *Materials and Methods:* Forty adult male albino rats were randomly allocated into four groups (n = 10 each): control, ivermectin-treated (0.6 mg/kg, orally on days 0, 1, 7, and 8), BLM-treated (single intratracheal dose of 5 mg/kg), and BLM- and ivermectin-treated. Lung tissues were collected for histopathological analysis and Mallory trichrome staining to assess collagen deposition. Mast cell (MC) infiltration was assessed using toluidine blue. Immunohistochemistry for α-SMA and Ki-67 was used to evaluate myofibroblast and cell proliferation. Oxidative stress parameters, including serum total antioxidant capacity, lung glutathione and lung nitric oxide were measured. *Results:* Ivermectin treatment markedly attenuated BLM-induced lung fibrosis, showing reduced collagen accumulation, restoration of alveolar architecture, and decreased inflammatory cell infiltration. Immunohistochemical evaluation revealed decreased expression of α-SMA and Ki-67, while biochemical analyses demonstrated improved oxidative stress markers. *Conclusions*: Ivermectin significantly mitigates BLM-induced pulmonary fibrosis in rats through modulation of inflammation, suppression of myofibroblast proliferation, and reduction in oxidative stress and collagen deposition. These findings highlight ivermectin as a potential candidate for the management of fibrotic lung diseases, warranting further mechanistic and clinical investigations.

## 1. Introduction

Pulmonary fibrosis (PF) is an interstitial lung disease that leads to parenchymal scarring and death due to respiratory failure. The interstitial PF induced by bleomycin (BLM) in rats comes from acute inflammatory damage [1]. The inflammatory process is a cascade of molecular events that involves the activation of alveolar epithelial cells, which secrete pro-fibrotic growth factors, mainly transforming growth factor-β (TGF-β), and the generation of fibroblasts and myofibroblasts, in turn, produces a large amount of extracellular matrix (ECM), such as collagen [2].

The criteria of diagnosis of interstitial PF depend on radiological and histopathological features. Epithelial–mesenchymal transition (EMT) illustrates the development of all fibrosing diseases. EMT is a biological mechanism through which the epithelial cells acquire the properties of mesenchymal cells and lose their normal criteria exhibiting migration and production of extracellular matrix. Migration, proliferation and activation of fibroblasts are induced by platelet-derived growth factor (PDGF), transforming growth factor beta-1 (TGF_1), and tumor necrosis factor (TNF), which are released by hyperactivated lung epithelial cells [1]. However, the sequence of events starts with inflammatory injuries of the alveolar epithelium, which leads to the release of pro-fibrotic growth factor, mainly TGF-β, and generation of fibroblasts and myofibroblasts, in turn, produces a large amount of ECM, such as collagen [3].The matrix deposition in lung parenchyma leads to the destruction of the alveolar architecture, loss of lung function, and eventually respiratory failure and death [4]. In recent years, a lot of studies have shown a close relationship between oxidative stress and pathogenesis of PF [2].

BLM is a glycopeptide antibiotic that possesses anti-neoplastic criteria. It is widely used to produce PF in animals, and it is one of the most frequently used models. BLM causes interstitial lung fibrosis in animals like human idiopathic PF. The pathological fibrotic changes depend on dose and route of administration [5]. It causes DNA damage through the production of free radicals and oxidative stress. This causes epithelial cell injury, activation of macrophages, differentiation of fibroblasts to myofibroblast and collagen deposition [6].

Ivermectin is a broad-spectrum antiparasitic agent with prominent antiviral and anti-inflammatory properties [7,8]. It exhibits antiviral effects against HIV-1, Dengue virus 1–4, and SARS-CoV-2 [9,10]. It has been found to inhibit various cancer diseases [11]. Interestingly, recent clinical investigations on COVID-19 revealed that patients treated with ivermectin had a reduced mortality rate than those not treated with it [12]. Also, COVID-19 patients who received standard-dose ivermectin (200 μg/kg) as hydroxychloroquine/azithromycin adjuvant therapy had considerably shorter hospital stays than patients who did not get ivermectin [13]. Ivermectin works as an anti-inflammatory agent by inhibiting the activation of T cells and suppressing the production of pro-inflammatory cytokines [7,14]. The therapeutic benefits of ivermectin in pulmonary fibrosis (PF) appear to result from its ability to inhibit the NLRP3 inflammasome, possibly through the downregulation of NF-κB and HIF-1α expression. It modulates TGF-β1 and pro-inflammatory cytokine levels to facilitate wound healing. TGF-β1 plays a pivotal role in the development of pulmonary fibrosis, as its signaling promotes myofibroblast activation, excessive collagen accumulation, and apoptosis of alveolar epithelial cells, as supported by previous studies [15,16]. It was reported that ivermectin induces acute toxicity as convulsions in association with its overdose in animals. In humans, it has been reported to induce neurological disorders by mutations in the human gene ABCB. It has been recorded that one of the side effects of ivermectin is decreasing platelet counts. These potential side effects of ivermectin limit its use at high doses in fibrosis [17,18].

The current study aimed to evaluate the possible effect of ivermectin on BLM-induced biochemical and histological changes in an experimental model of PF.

## 2. Materials and Methods

### 2.1. Drugs

BLM (Bleocip 15 IU; Cipla, Mumbai, India) was prepared in sterile saline to yield a 5 mg/mL solution.

Ivermectin (Iverzine; Unipharma, Cairo, Egypt) was prepared in distilled water at 0.6 mg/mL. Thiopental sodium was obtained from the Egyptian International Pharmaceutical Industries Company (E.I.P.I.CO., Tens of Ramadan, Egypt). All additional reagents were analytical grade.

### 2.2. Animals

#### 2.2.1. Animals Used in the Experiment

Forty adult male albino rats (200–220 g) were sourced from the Experimental Animal Research Unit, Faculty of Medicine, Ain Shams University (Cairo, Egypt). Animals were maintained under controlled environmental conditions (temperature (23 ± 2) C relative humidity 55±2%) on a 12 h light/dark schedule, with unrestricted access to standard chow and water. Sample size calculation: Power calculation was used due to availability of knowledge about the effect sample size and standard deviation from previous experiments. The sample size was calculated using PASS11 version 11.0.10, based on the minimal difference between groups regarding the mean level of tissue grading. With alpha level set at 0.05 and beta error set at 0.1 (power 90%), a total sample size of 40 rats—10 per group—was calculated [15]. This study was carried out in compliance with the ARRIVE 2.0 guidelines for the reporting of animal experiments. Ethical clearance was granted by the Research Ethics Committee (REC) of the Faculty of Medicine, Helwan University, Cairo, Egypt, under serial number 40-2025.

#### 2.2.2. Induction of Pulmonary Fibrosis

Pulmonary fibrosis was elicited by a single intratracheal instillation of BLM (5 mg/kg; dosing volume 1 mL/kg), in accordance with established protocols. Anesthesia was achieved with intraperitoneal thiopental sodium (50 mg/kg) as per prior description.

#### 2.2.3. Experimental Design

After baseline weighing, the forty rats were randomized into four groups (n = 10 each):

Group I (Control): Intratracheal saline (1 mL/kg); served as the normal control.

Group II (Ivermectin): Oral ivermectin 0.6 mg/kg once daily on days 0, 1, 7, and 8, following a dosing schedule reported in the clinical context [15].

Group III (BLM): Single intratracheal BLM dose (5 mg/kg) as previously described [19].

Group IV (BLM + Ivermectin): Combined treatment with intratracheal BLM (5 mg/kg, single dose) plus oral ivermectin (0.6 mg/kg) administered once daily on days 0, 1, 7, and 8.

### 2.3. Tissue Processing, Histological and Immunohistochemical Analysis

Animals were euthanized by cervical dislocation; both lungs were excised, rinsed in cold saline, and blotted dry, and the right lung was allocated for histological and immunohistochemical evaluation.

#### 2.3.1. Light Microscopic Analysis

Paraffin blocks were sectioned at 4–5 μm. Sections were rehydrated and stained sequentially with hematoxylin and eosin (H&E), Mallory’s trichrome, and toluidine blue according to standard histological procedures and referenced manual [20].

#### 2.3.2. Immunohistochemical Analysis

α-Smooth muscle actin (α-SMA): α-SMA was used to identify myofibroblasts and smooth muscle cells as indicators of fibrosis and tissue remodeling. Endogenous peroxidase activity was quenched with 3% hydrogen peroxide in deionised water for 30 min at room temperature. Sections were incubated with a monoclonal anti-SMA antibody IgG (Lab Vision NeoMarkers, Fremont, CA, USA; Cat. No. 1-GE002-07), yielding a brown cytoplasmic stain in positive cells. Colon sections served as positive controls (smooth muscle positivity), and negative controls were processed in parallel with the primary antibody omitted [20].

Ki-67: It is a rabbit monoclonal antibody IgG (Lab Vision Epredia™, Kalamazoo, MI, USA, Cat noRM9106S0). It is a nuclear marker of cellular proliferation and was evaluated on deparaffinized sections. The positive control was the tonsil. After blocking with normal goat serum for 1 h (and quenching endogenous peroxidase as per routine), sections were incubated overnight at 4 °C with anti–Ki-67 antibody (1:1000), following standard immunohistochemical practice [20].

### 2.4. Sample Collection and Biochemical Analyses

At day 14 post BLM, retro-orbital blood was collected under Na thiopental anesthesia (50 mg/kg, intraperitoneal) using heparinized microcapillaries (Opti Lab, Berlin, Germany). Serum was isolated by centrifugation (4000 rpm, 10 min, −4 °C; Heraeus Biofuge, Berlin, Germany).

Total antioxidant capacity (TAC): Serum TAC was quantified using commercial kits (Biodiagnostic, Giza, Egypt) according to the supplier’s instruction [21].

Reduced glutathione (GSH) and nitric oxide (NO): Left lung lobes were rinsed and homogenized to 10% (*w*/*v*) in ice-cold 1.15% KCl (pH 7). Homogenates were centrifuged (2000 rpm, 15 min, 4 °C), and supernatants were immediately assayed for GSH and NO using Biodiagnostic kits (Giza, Egypt), following manufacturer protocols aligned with the established method [22].

### 2.5. Morphometric and Digital Image Analysis

Quantitative image analysis was performed using Leica LAS X 2D s/w software with the measurement module. Ten representative fields per slide for each experimental group were captured for assessment. The following parameters were obtained:

1. Interalveolar septal thickness (H&E; μm)
2. Area percentage of Mallory’s trichrome staining (collagen) at ×40
3. Area percentage of α-SMA-positive immunoreactivity at ×40
4. Number of Ki-67-positive nuclei (immunostained at ×40)

### 2.6. Statistical Analysis

Morphometric and biochemical data from the four groups were analyzed using SPSS (version 23) (SPSS Inc., Chicago, IL, USA). The data were summarized using mean ± standard deviation (SD). Statistical significance was set at *p* ≤ 0.05. One-way analysis of variance (ANOVA) was performed, followed by Tukey’s post hoc test = LSD for multiple comparisons [23].

## 3. Results

### 3.1. Histology

#### 3.1.1. Hematoxylin and Eosin

**Figure 1:**
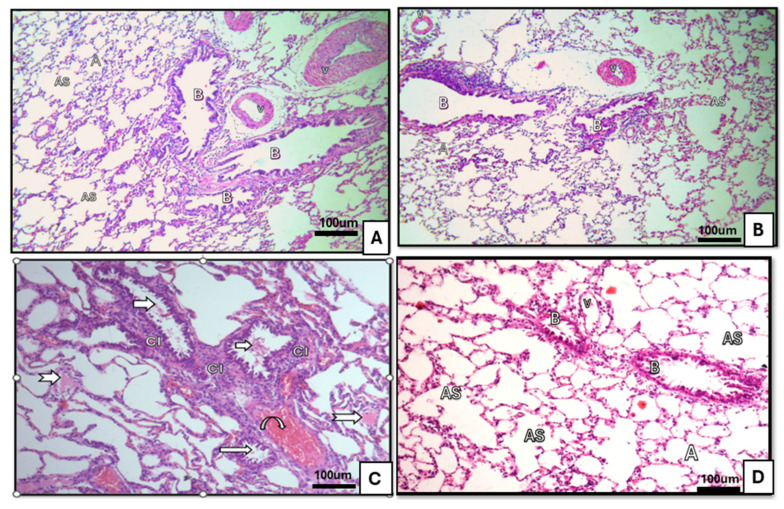
Sections of lung tissue of albino rats stained by (Hx&E ×40). (**A**) Control group showing bronchioles (B), alveolar sacs (AS), alveoli (A) with thin interalveolar septum, and a blood vessel (V). (**B**) The ivermectin-treated group was similar to the control. (**C**) BLM-treated group showing bronchioles (arrows): distorted bronchiolar epithelium with luminal debris and mononuclear cell infiltration (CI), acidophilic homogenous hyaline cast within bronchioles (bifurcated arrows), and dilated blood vessel (curved arrow) with extravasated blood. (**D**) BLM- and ivermectin-treated group showing multiple alveolar sacs (AS), normal thin blood vessel (v), and alveoli (A) and bronchiole (B) mostly appearing normal.

**Figure 2:**
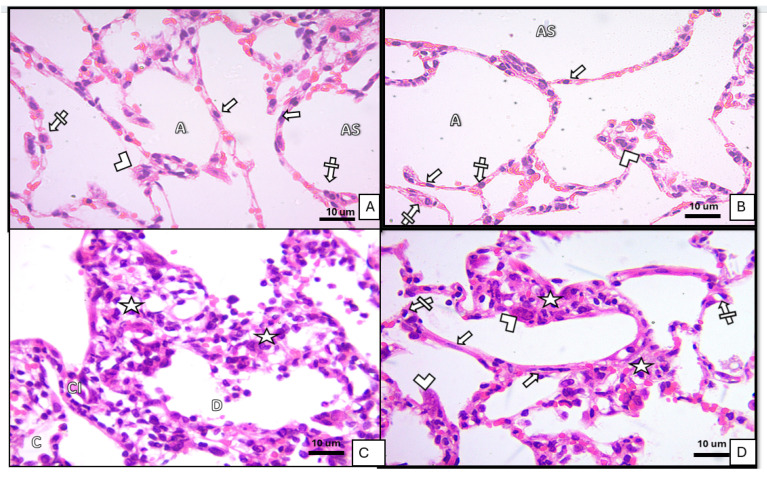
Sections of lung tissue of albino rats stained by (Hx&E ×400) showing intra-alveolar septa. (**A**) Control group showing patent alveoli (A) and alveolar sacs (AS). Alveoli are lined by type I pneumocytes (arrows), type II pneumocytes (crossed arrows), and alveolar macrophages (arrowheads). (**B**) Ivermectin-treated group showing similar histology as the control group. (**C**) BLM-treated group showing some thickened interalveolar septa (asterisk) with mononuclear cell infiltration (CI), and other alveolar septa are distorted (D). There was an area of alveolar collapse (C). (**D**) BLM- and ivermectin-treated group showing some recovery in intra-alveolar septa; some septa were thick with infiltrating mononuclear cells (asterisk); most of the thin-walled alveoli were lined with pneumocyte type I (arrow); and cuboidal cells, pneumocyte type II (crossed arrow) and the alveolar macrophage also appear (arrowhead).

**Figure 3:**
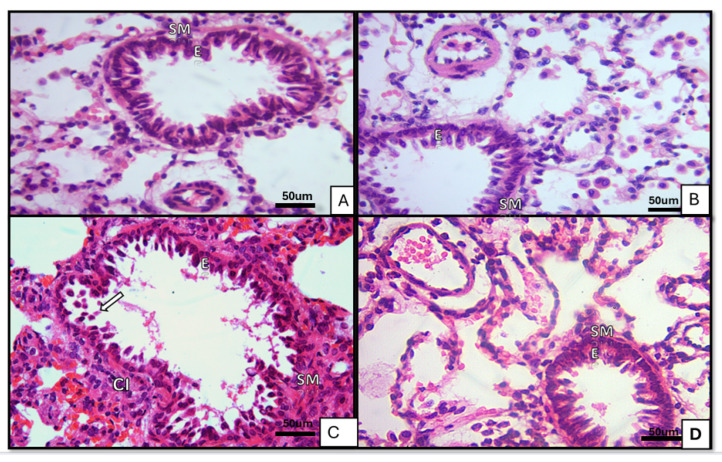
Sections of lung tissue of albino rats stained by (Hx&E ×200) showing bronchioles. (**A**) Control group: bronchiolar mucosa lined by pseudostratified columnar cells (E) with spiral layer of smooth muscle (SM). (**B**) Ivermectin-treated group was similar to the control. (**C**) BLM-treated group was lined by pseudostriated epithelium (E) with luminal cellular debris (arrows) and mononuclear cell infiltration (CI) with hyperplastic smooth muscle (SM). (**D**) BLM- and ivermectin-treated group similar to the control group.

1. Control and ivermectin groups: Bronchioles exhibited mucosa lined by pseudostratified columnar epithelium with a surrounding spiral layer of smooth muscle and an adventitia composed of areolar connective tissue. Alveolar sacs and alveoli were patent and lined by type I and type II pneumocytes, with thin interalveolar septa and normal blood capillaries. The ivermectin group was comparable to controls Figure 1A,B, Figure 2A,B and Figure 3A,B.
2. BLM group: Bronchiolar epithelium appeared distorted with luminal cellular debris, peribronchiolar mononuclear infiltrates and homogenous eosinophilic hyaline cast. Blood capillaries were dilated and congested. Alveoli displayed a mixed pattern of collapse and overdistension Figure 1C, Figure 2C and Figure 3C.
3. BLM+ ivermectin group: Lung architecture showed partial preservation, with mild interalveolar septal thickening accompanied by scattered mononuclear cells. Most alveoli appeared normal, with occasional mild dilatation; type I pneumocytes were predominant, with identifiable cuboidal type II cells and visible alveolar macrophages Figure 1D, Figure 2D and Figure 3D.

#### 3.1.2. Mallory Trichrome and Tolidine Blue

**Figure 4:**
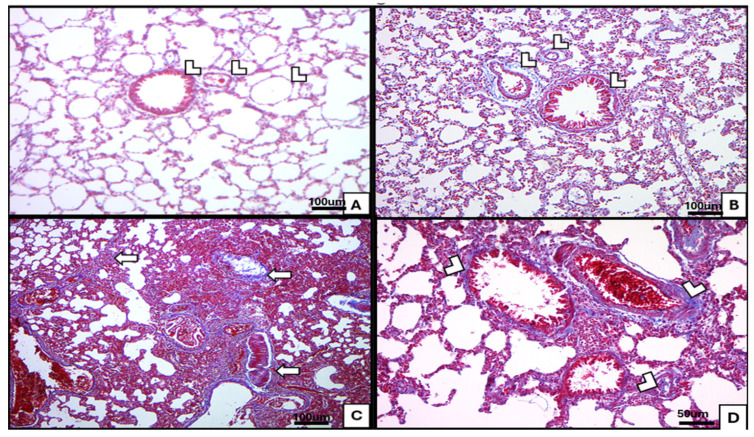
Sections from lung tissue stained by Mallory trichrome (×40& ×200) (special stain for collagen fibers). (**A**) Section of the lung of an albino rat (control group), showing fine collagen fibers within the lung interstitium and the adventitia of bronchioles (arrowhead). (**B**) Ivermectin-treated group similar to the control group. (**C**) Section of the lung of the BLM-treated group, showing extensive collagen fiber deposition in the lung interstitium and around blood vessels (arrows). (**D**) BLM- and ivermectin-treated group showing moderate collagen fibers in the lung interstitium around bronchioles and blood vessels (arrowhead).

**Figure 5:**
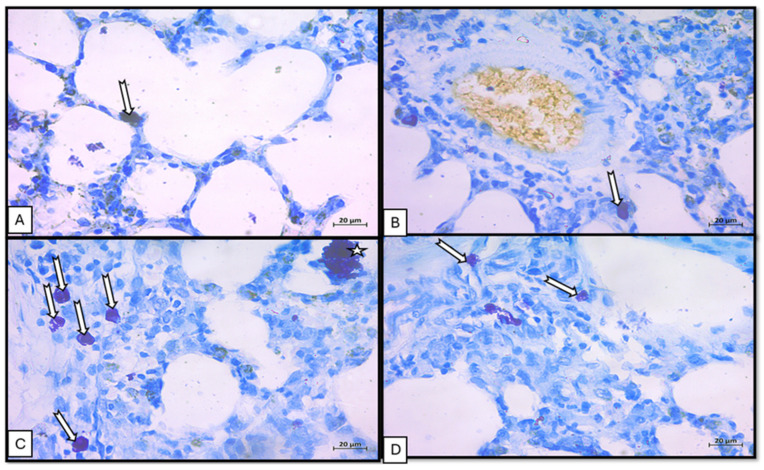
Sections of lung tissue stained with (toluidine blue ×100) (special stain for granulated MCs). (**A**) Sections of lung control group showed granulated MCs in the connective tissue in interalveolar septa (bifurcated arrow). (**B**) Sections of lung from ivermectin-treated group showed MCs in the interalveolar septa (bifurcated arrow). (**C**) BLM-treated group showed an increase in MCs in interalveolar septa (bifurcated arrows) with large granulated MCs (asterisk). (**D**) BLM- and ivermectin-treated group showed few granulated MCs in the interalveolar space (bifurcated arrow).

1. Mallory’s trichrome (collagen): Control group exhibited fine collagen fibers in the interstitium and within bronchiolar adventitia ivermectin group resembled control Figure 4A,B. BLM exposure produced extensive collagen deposition throughout the interstitium and around vessels Figure 4C. Combined BLM and ivermectin treatment showed moderate collagen fibers in the lung interstitium around bronchioles and blood vessels Figure 4D.
2. Toluidine blue (MCs): Toluidine blue staining highlighted metachromatic violet granules in MCs. Control group showed scattered granulated MCs within interalveolar connective tissue; ivermectin group had a similar distribution, including cells near vessels Figure 5A,B. BLM increased MC numbers in interalveolar septa and included larger, prominently granulated cells Figure 5C. The combination group showed fewer granulated MCs within interalveolar spaces Figure 5D.

#### 3.1.3. Immunohistochemistry

**Figure 6:**
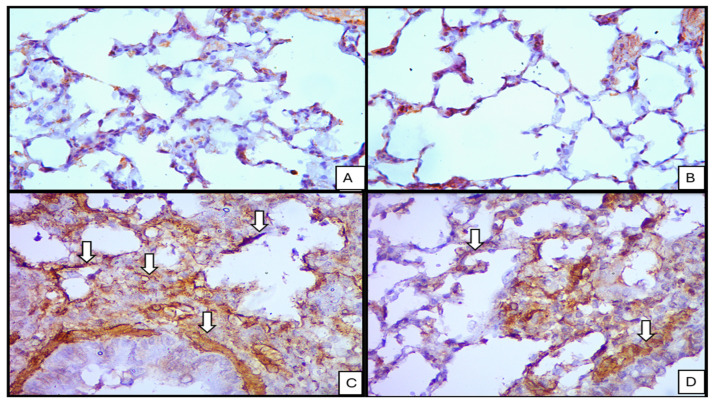
Sections from lung tissue illustrate immunohistochemistry for (α-smooth muscle actin · 400) (a marker for myofibroblasts and smooth muscle cells) showing: (**A**) control lung, showing minimal immune reaction; (**B**) ivermectin-treated lung, also showing minimal immune reaction; (**C**) BLM-treated lung showing strong positive cytoplasmic immune reaction (arrows); (**D**) BLM- and ivermectin-treated tissue showing moderate cytoplasmic positivity (arrows).

**Figure 7:**
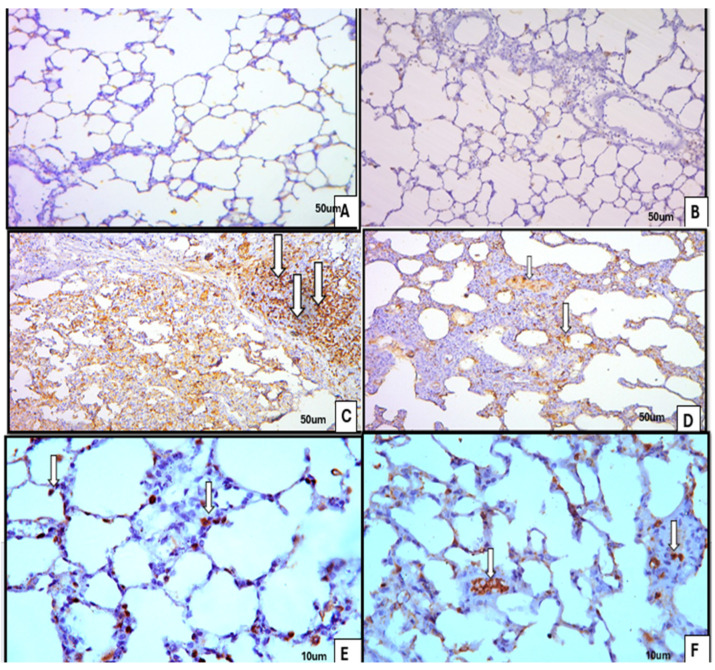
Sections from lung tissue illustrate immunohistochemistry for Ki-67 antibody (proliferation marker) (×200, ×400): (**A**) control group, showing minimal immune reaction; (**B**) ivermectin-treated group, also showing minimal immune reaction; (**C**,**E**) BLM-treated group, showing strong positive nuclear (arrows) immune reaction; and (**D**,**F**) BLM- and ivermectin-treated group, showing moderately positive nuclear immune reaction (arrows).

1. α-SMA: Control and ivermectin group lungs showed minimal α-SMA immunoreactivity beyond expected smooth muscle structures. Following BLM, strong cytoplasmic α-SMA expression was evident in myofibroblast-like cells and in the smooth muscle of bronchioles. The combination treatment reduced α-SMA labeling to moderate levels in both parenchymal cells and bronchiolar smooth muscle Figure 6.
2. Ki-67: In control and ivermectin groups lung numbers of Ki-67-positive nuclei were negligible. BLM-treated lungs displayed strong nuclear positivity consistent with increased proliferative activity. The BLM + ivermectin group demonstrated a moderate number of Ki-67-positive nuclei Figure 7.

### 3.2. Blood Chemistry

The biochemical results of serum TAC, lung GSH, and lung NO levels across all different experimental groups are presented in Table 1. There was a statistically significant decrease in serum TAC and lung GSH and an increase in the lung NO levels in the BLM group in relation to the other groups, indicating significant oxidative stress in the BLM group. There was a statistically significant increase in serum TAC and lung GSH and a decrease in the lung NO levels in the BLM and ivermectin group in relation to the BLM group, indicating significant improvement of the oxidative stress in comparison to the BLM group.

### 3.3. Morphometric Analysis

- BLM group exhibited severe pathological changes as expressed in Table 2:
  ○ Thickened alveolar septa (indicating fibrosis or inflammation). It showed a significant increase (12.43 ± 1.78 µm) compared to all other groups.
  ○ Increased collagen deposition (MT staining) of (5.00 ± 0.84%) exhibited significantly higher fibrosis than all other groups.
  ○ Elevated myofibroblast activity (α-SMA) (23.17 ± 4.62%) had markedly higher α-SMA, suggesting increased myofibroblast activation.
  ○ Ki-67-positive nuclei displayed higher cell proliferation with a significant increase in relation to other groups (13.33 ± 1.37%).
- BLM and ivermectin group showed significant improvement, suggesting a protective or therapeutic effect compared to BLM group. The thickened alveolar septa showed that (3.07 ± 0.36 µm) had a significant decrease compared to BLM group. Collagen deposition (MT staining) showed a significant reduction compared to BLM group. Myofibroblast activity showed a significant decrease compared to BLM group. Cellular proliferation showed a significant reduction, indicating suppressed proliferation compared to BLM group, with a significant difference indicating some improvement. Also, there was a significant increase in the number of Ki67-positive nuclei in relation to control groups (I&II), indicating that this treated group did not return to the baseline level as expressed in Table 2.

## 4. Discussion

Severe COVID-19 predominantly affects the lungs and, in critical illness, progresses to acute respiratory distress syndrome, causing extensive parenchymal injury; subsequent repair can follow a fibrotic change that culminates in PF [24].

BLM, a chemotherapeutic antibiotic derived from Streptomyces verticillus, is widely used in the treatment of various malignancies, including Hodgkin’s and non-Hodgkin lymphomas, testicular cancer, and squamous cell carcinomas [25]. However, its clinical use is limited due to its well-documented pulmonary toxicity, including inflammation and fibrosis. Cytokines and chemokines play an important role in the induction of PF caused by BLM. Cytokines like interleukin (IL)-1α, IL-1β, TNF-α, TGF-β, and PDGF play their role through the mobilization, activation, and proliferation of fibroblasts, macrophages, and myofibroblasts, respectively, and they can exhibit significant pro-fibrotic activity. Chemokines play their role through recruiting and activating mononuclear cells, enhancing macrophage mobilization to the lungs and controlling the mobilization of fibrocytes and monocytes to fibrotic sites in the lungs [6].

PF was induced using BLM, which led to pronounced structural and biochemical alterations in lung tissue, in line with previous findings [15]. BLM exerts its toxic effects by inducing oxidative stress through the generation of reactive oxygen species (ROS) via its interaction with Fe^2+^ and O_2_, resulting in direct damage to bronchiolar epithelial cells [26]. This mechanism was further supported by our biochemical findings, including depletion of serum TAC and lung GSH in BLM-treated rats further confirming oxidative-stress-mediated injury [27,28]. Additionally, inducible nitric oxide synthase (iNOS) expression and NO levels were elevated, which can react with superoxide to form peroxynitrite—a potent pro-oxidant contributing to tissue injury and fibrosis [28].

Vascular changes observed, such as capillary congestion and thickening, were consistent with reports attributing such changes to ROS-induced vascular remodeling and increased capillary permeability [29]. Histologically, BLM caused thickening of alveolar septa, inflammatory cell infiltration, and ECM deposition [30]. Damage to type I pneumocytes led to alveolar collapse and impaired gas exchange, while type II pneumocyte hyperplasia and metaplastic changes were evident as part of the regenerative response [31,32]. These findings were statistically confirmed and aligned with prior observations [33].

Mallory’s trichrome staining confirmed robust collagen deposition in septa, peribronchiolar, and perivascular regions [34]. The fibrotic cascade is attributed to oxidative stress, pro-inflammatory cytokines (TNF-α, IL-1β, IL-6), and TGF-β1-driven fibroblast activation, epithelial–mesenchymal transition, and myofibroblast differentiation [35].

Toluidine blue staining showed a marked increase in MCs following BLM administration. These cells contribute to fibrosis through the release of histamine, which stimulates fibroblast proliferation, and renin, which enhances local angiotensin II production, thus promoting fibrogenesis [36]. Although toluidine blue is not specific to macrophages, these cells also play a central role in fibrosis by releasing pro-inflammatory and pro-fibrotic mediators, including TGF-β, correlating with the extent of ECM deposition [22].

Our results further demonstrated a significant increase in α-smooth muscle actin (α-SMA) expression in BLM-treated lungs, indicating myofibroblast activation, particularly within fibrotic foci [37,38]. This upregulation is strongly associated with TGF-β signaling, which promotes fibroblast-to-myofibroblast transition and enhances ECM production [39]. Moreover, the BLM-treated lungs exhibited a higher number of Ki-67-positive nuclei, reflecting enhanced cellular proliferation. The elevated Ki-67 expression indicates a dysregulated proliferative response of the alveolar epithelium and stromal cells, which may play a role in the ensuing degenerative and fibrotic alterations [40].

In this study, ivermectin mitigated pathology in a rat model of BLM-induced pulmonary fibrosis, supporting its proposed anti-inflammatory and antifibrotic activity. Rats treated with both BLM and ivermectin exhibited significant histological and biochemical improvements. Lung sections from this group showed restored alveolar architecture, reduced septal thickening, and minimal inflammatory infiltration, resembling normal control lungs. These results were consistent with previous reports [41]. Mallory’s trichrome staining demonstrated a significant reduction in collagen deposition in the ivermectin co-treatment group, further supporting its antifibrotic potential. Ki-67 expression was reduced following ivermectin treatment, likely as a result of its anti-inflammatory effects, decreased collagen accumulation, and inhibition of cellular proliferation, indicating a corresponding suppression of fibroblast activity within the lung tissue [15]. Biochemically, the BLM + ivermectin group showed significantly reduced NO levels and restoration of GSH and TAC, suggesting mitigation of oxidative stress. Immunohistochemically, there was a marked reduction in α-SMA expression, indicating decreased myofibroblast differentiation and fibrosis severity [42]. Ivermectin’s impact on MC numbers was also notable, with a significant decrease observed in the co-treated group compared to BLM-only rats. This may be attributed to ivermectin’s ability to inhibit the NLRP3 inflammasome, thereby reducing levels of IL-1β and TGF-β1, both known to activate MCs [41]. MCs were found in large numbers in cases of pulmonary fibrosis. MCs appeared to share in the initial lung injury induced by BLM through the release of the MC protease 4, histamine, and renin angiotensin II. So, MCs play an important, pro-fibrotic role in BLM-induced lung fibrosis by initiating inflammation and activating fibroblasts, collagen deposition, TGF-β activation, and fibrosis progression [43].

Mechanistically, ivermectin exerts anti-inflammatory effects by inhibiting the NF-κB pathway, leading to reduced production of pro-inflammatory cytokines such as TNF-α, IL-1β, and IL-6. Its antifibrotic action has been linked to downregulation of the TGF-β/Smad pathway, inhibition of fibroblast proliferation and activation, and attenuation of oxidative stress [44]. In pulmonary fibrosis and lung injury, the relation between the mechanisms of action of BLM and ivermectin is an antagonistic one, where ivermectin acts as a therapeutic agent that converses the damage caused by BLM [41].

### Study Limitations

1. The parameters for Gamma-glutamyl transferase (γ-GT) and alkaline phosphatase were not measured.
2. Lack of assessment of functional lung parameters (e.g., lung compliance, forced vital capacity and total lung capacity).

## 5. Conclusions

Collectively, our findings demonstrate that ivermectin significantly ameliorates BLM-induced pulmonary fibrosis through its combined anti-inflammatory, antioxidant, and antifibrotic actions. These results suggest that ivermectin holds promise as an adjuvant therapeutic agent to mitigate pulmonary toxicity associated with BLM treatment. Nevertheless, further mechanistic and clinical studies are essential to validate these effects and clarify the molecular pathways involved in ivermectin’s protective actions.

## Figures and Tables

**Table 1:** The measured levels of serum TCA, lung GSH, and lung NO in all studied groups.

	Serum TAC	Lung GSH	Lung NO
Group I Means ± SD	1.30 ± 0.06	2.00 ± 0.14	1.29 ± 0.01
Group II Means ± SD	1.23 ± 0.01	1.96 ± 0.23	1.32 ± 0.31
Group III Means ± SD	0.58 ± 0.12 ^a^	0.77 ± 0.24 ^a^	3.22 ± 0.38 ^c^
Group IV Means ± SD	1.19 ± 0.07 ^b^	1.82 ± 0.17 ^b^	1.60 ± 0.38 ^d^

All data are expressed as mean ± SD. A significant difference is considered when *p* value ≤ 0.05. a = Significant decrease concerning group I&II&IV; b = Significant increase concerning group III; c = Significant increase in relation to group I&II&IV; d = Significant decrease concerning group III.

**Table 2:** Thickness of interalveolar septa (IAS) in H&E-stained sections (µm), area percentage of Mallory trichrome staining (MT), area percentage of α-smooth muscle actin immunostaining, and number of Ki-67-positive nuclei in all studied groups.

	IAS (µm)	ML (X40)	α-Smooth Muscle Actin (X40)	ki 67 (X40)
Group I Means ± SD	2.17 ± 0.41	0.80 ± 0.55	3.28 ± 1.40	2.00 ± 0.63
Group II Means ± SD	2.70 ± 0.33	0.50 ± 0.53	2.92 ± 0.60	2.00 ± 0.63
Group III Means ± SD	12.43 ± 1.78 ^a^	5.00 ± 0.84 ^a^	23.17 ± 4.62 ^a^	13.33 ± 1.37 ^a^
Group IV Means ± SD	3.07 ± 0.36 ^b^	1.00 ± 0.09 ^b^	3.39 ± 0.84 ^b^	3.17 ± 0.75 ^b,c^

All data are expressed as mean ± SD. A significant difference is considered when *p* value ≤ 0.05. a = Significant increase concerning group I&II&VI; b = Significant decrease concerning group III; c = Significant increase concerning group I&II.

## Data Availability

All data supporting the findings of this study are available upon reasonable request.

## References

[1] Salton F., Ruaro B., Confalonieri P., Confalonieri M. (2020). Epithelial–mesenchymal transition: A major pathogenic driver in idiopathic pulmonary fibrosis?. Medicina.

[2] Xu D., Wang Q., Lyu M., Huang C., Yuan X., Chen X., Huang Y. (2026). The Mechanism of Oxidative Stress in Pulmonary Fibrosis and Research Progress. Antioxidants.

[3] Baratella E., Ruaro B., Giudici F., Wade B., Santagiuliana M., Salton F., Confalonieri P., Simbolo M., Scarpa A., Tollot S. (2021). Evaluation of correlations between genetic variants and high-resolution computed tomography patterns in idiopathic pulmonary fibrosis. Diagnostics.

[4] Wang J., Li K., Hao D., Li X., Zhu Y., Yu H., Chen H. (2024). Pulmonary fibrosis: Pathogenesis and therapeutic strategies. MedComm.

[5] Wu S., Driver I., Luo M., Miyazaki H., Shambhu S., Popov D., Yang L., Wang J., Ma J., Guo J. (2025). Ferret model of bleomycin-induced lung injury shares features of human idiopathic pulmonary fibrosis. NPJ Regen. Med..

[6] Ishida Y., Kuninaka Y., Mukaida N., Kondo T. (2023). Immune mechanisms of pulmonary fibrosis with bleomycin. Int. J. Mol. Sci..

[7] Steinhoff M., Vocanson M., Voegel J.J., Hacini-Rachinel F., Schäfer G. (2016). Topical ivermectin 10 mg/g and oral doxycycline 40 mg modified-release: Current evidence on the complementary use of anti-inflammatory rosacea treatments. Adv. Ther..

[8] Mansour S.M., Shamma R.N., Ahmed K.A., Sabry N.A., Esmat G., Mahmoud A.A., Maged A. (2021). Safety of inhaled ivermectin as a repurposed direct drug for treatment of COVID-19: A preclinical tolerance study. Int. Immunopharmacol..

[9] Martin A.J., Jans D.A. (2021). Antivirals that target the host IMPα/β1-virus interface. Biochem. Soc. Trans..

[10] Martin R.J., Robertson A.P., Choudhary S. (2021). Ivermectin: An anthelmintic, an insecticide, and much more. Trends Parasitol..

[11] Li N., Zhan X. (2020). Anti-parasite drug ivermectin can suppress ovarian cancer by regulating lncRNA-EIF4A3-mRNA axes. EPMA J..

[12] Rajter J.C., Sherman M.S., Fatteh N., Vogel F., Sacks J., Rajter J.J. (2021). Use of ivermectin is associated with lower mortality in hospitalized patients with coronavirus disease 2019: The ivermectin in COVID nineteen study. Chest.

[13] Gorial F.I., Mashhadani S., Sayaly H.M., Dakhil B.D., AlMashhadani M.M., Aljabory A.M., Abbas H.M., Ghanim M., Rasheed J.I. (2020). Effectiveness of ivermectin as add-on therapy in COVID-19 management (pilot trial). medRxiv.

[14] Kaur B., Blavo C., Parmar M.S. (2024). Ivermectin: A multifaceted drug with a potential beyond anti-parasitic therapy. Cureus.

[15] Abd-Elmawla M.A., Ghaiad H.R., Gad E.S., Ahmed K.A., Abdelmonem M. (2023). Suppression of NLRP3 inflammasome by ivermectin ameliorates bleomycin-induced pulmonary fibrosis. J. Zhejiang Univ. Sci. B.

[16] Han Y., Jiang M., He R., Lv X., Liao X., He Y., Zhang F., Long L., Jiang G., Peng Z. (2021). Mefunidone ameliorates bleomycin-induced pulmonary fibrosis in mice. Front. Pharmacol..

[17] Abou El-Fetouh M.S., Elseddawy N.M., Abdelsamia H.M. (2024). Insights on the therapeutic use of ivermectin: Mechanism of action and histopathological effects. J. Adv. Vet. Res..

[18] Ashour D.S. (2019). Ivermectin: From theory to clinical application. Int. J. Antimicrob. Agents.

[19] Wang Z., Li X., Chen H., Han L., Ji X., Wang Q., Wei L., Miu Y., Wang J., Mao J. (2021). Resveratrol alleviates bleomycin-induced pulmonary fibrosis via suppressing HIF-1α and NF-κB expression. Aging.

[20] Suvarna K.S., Layton C., Bancroft J.D. (2018). Bancroft’s Theory and Practice of Histological Techniques.

[21] Blanco A., Fernandes R., Guimarães G.R., Rigatto K. (2025). Alamandine reduces oxidative stress and preserves the epithelium in BLM-induced pulmonary fibrosis. Eur. J. Pharmacol..

[22] Comeglio P., Filippi S., Sarchielli E., Morelli A., Cellai I., Corno C., Pini A., Adorini L., Vannelli G.B., Maggi M. (2019). Therapeutic effects of obeticholic acid (OCA) treatment in a bleomycin-induced pulmonary fibrosis rat model. J. Endocrinol. Investig..

[23] Field A. (2024). Discovering Statistics Using IBM SPSS Statistics.

[24] Najjar-Debbiny R., Barnett-Griness O., Khoury J., Gronich N., Weber G., Adir Y., Shteinberg M., Shneir S., Sharma L., Saliba W. (2023). Association between COVID-19 infection and pulmonary fibrosis: A nested case-control study. Am. J. Med..

[25] Park J.K., Coffey N.J., Bodine S.P., Zawatsky C.N., Jay L., Gahl W.A., Kunos G., Gochuico B.R., Malicdan M.C.V., Cinar R. (2020). Bleomycin induces drug efflux in lungs. A pitfall for pharmacological studies of pulmonary fibrosis. Am. J. Respir. Cell Mol. Biol..

[26] Dossett C., Yost K., Lau C., Shamsid-Deen N. (2022). A Case of Progressive Bleomycin Lung Toxicity Refractory to Steroid Therapy. Southwest J. Pulm. Crit. Care Sleep.

[27] Estornut C., Milara J., Bayarri M.A., Belhadj N., Cortijo J. (2022). Targeting oxidative stress as a therapeutic approach for idiopathic pulmonary fibrosis. Front. Pharmacol..

[28] Chen C., Yun X.J., Liu L., Guo H., Liu L.F., Chen X.L. (2017). Exogenous nitric oxide enhances the prophylactic effect of aminoguanidine, a preferred iNOS inhibitor, on bleomycin-induced fibrosis in the lung: Implications for the direct roles of the NO molecule in vivo. Nitric Oxide.

[29] Rago F., Melo E.M., Kraemer L., Galvão I., Cassali G.D., Santos R.A.S., Russo R.C., Teixeira M.M. (2019). Effect of preventive or therapeutic treatment with angiotensin 1–7 in a model of bleomycin-induced lung fibrosis in mice. J. Leukoc. Biol..

[30] Tian S.L., Yang Y., Liu X.L., Xu Q.B. (2018). Emodin attenuates bleomycin-induced pulmonary fibrosis via anti-inflammatory and anti-oxidative activities in rats. Med. Sci. Monit..

[31] Kato S., Inui N., Hakamata A., Suzuki Y., Enomoto N., Fujisawa T., Nakamura Y., Watanabe H., Suda T. (2018). Changes in pulmonary endothelial cell properties during bleomycin-induced pulmonary fibrosis. Respir. Res..

[32] Lopez-Rodriguez E., Gay-Jordi G., Knudsen L., Ochs M., Serrano-Mollar A. (2021). Improved alveolar dynamics and structure after alveolar epithelial type II cell transplantation in bleomycin induced lung fibrosis. Front. Med..

[33] Tang H., Gao L., Mao J., He H., Liu J., Cai X., Lin H., Wu T. (2016). Salidroside protects against bleomycin-induced pulmonary fibrosis: Activation of Nrf2-antioxidant signaling, and inhibition of NF-κB and TGF-β1/Smad-2/-3 pathways. Cell Stress Chaperones.

[34] El-Bassouny D.R., Omar N.M., Khalaf H.A., Abd Al-Salam R.A. (2021). Role of nuclear factor-kappa B in bleomycin induced pulmonary fibrosis and the probable alleviating role of ginsenoside: Histological, immunohistochemical, and biochemical study. Anat. Cell Biol..

[35] Li J., Wei Q., Song K., Wang Y., Yang Y., Li M., Yu J., Su G., Peng L., Fu B. (2023). Tangeretin attenuates bleomycin-induced pulmonary fibrosis by inhibiting epithelial-mesenchymal transition via the PI3K/Akt pathway. Front. Pharmacol..

[36] El-Mohandes E.M., Moustafa A.M., Khalaf H.A., Hassan Y.F. (2017). The role of mast cells and macrophages in amiodarone induced pulmonary fibrosis and the possible attenuating role of atorvastatin. Biotech. Histochem..

[37] Zeng Q., Zhou T.-T., Huang W.-J., Huang X.-T., Huang L., Zhang X.-H., Sang X.-X., Luo Y.-Y., Tian Y.-M., Wu B. (2023). Asarinin attenuates bleomycin-induced pulmonary fibrosis by activating PPARγ. Sci. Rep..

[38] Sorkhdini P., Klubock-Shukla K., Sheth S., Yang D., Yang A.X., Norbrun C., Introne W.J., Gochuico B.R., Zhou Y. (2024). Type 2 innate immunity promotes the development of pulmonary fibrosis in Hermansky-Pudlak syndrome. JCI Insight.

[39] Hsu Y.A., Chang C.Y., Lan J.L., Li J.P., Lin H.J., Chen C.S., Wan L., Liu F.-T. (2020). Amelioration of bleomycin-induced pulmonary fibrosis via TGF-β-induced Smad and non-Smad signaling pathways in galectin-9-deficient mice and fibroblast cells. J. Biomed. Sci..

[40] Avezova D.B., Tilavov T.B.U., Khasanova D.A. (2026). KI-67 Immunohistochemical Expression in Lung Tissue of Rats under Chronic Renal Failure. Ibnosina J. Med. Biomed. Sci..

[41] Habibi Razi F., Mohammad Jafari R., Manavi M.A., Sheibani M., Rashidian A., Tavangar S.M., Beighmohammadi M.T., Dehpour A.R. (2024). Ivermectin ameliorates bleomycin-induced lung fibrosis in male rats by inhibiting the inflammation and oxidative stress. Immunopharmacol. Immunotoxicol..

[42] Elgendy M.S., Elsayed A., Eldosokey D.E., Abd Elmaqsoud A.K. (2021). Histological and immunohistochemical study to evaluate the effects of metformin versus green tea extracts on bleomycin induced lung injury in adult male albino rats. Egypt. J. Histol..

[43] Bhattacharyya A., Yadav P., Bhattacharya M. (2025). Myeloid-Mesenchymal Crosstalk in Lung Fibrosis. Compr. Physiol..

[44] Gao X., Xuan Y., Zhou Z., Chen C., Wang D.W., Wen Z. (2024). Ivermectin ameliorates acute myocarditis via the inhibition of importin-mediated nuclear translocation of NF-κB/p65. Int. Immunopharmacol..

